# Estimates of Outcomes Up to Ten Years after Stroke: Analysis from the Prospective South London Stroke Register

**DOI:** 10.1371/journal.pmed.1001033

**Published:** 2011-05-17

**Authors:** Charles D. A. Wolfe, Siobhan L. Crichton, Peter U. Heuschmann, Christopher J. McKevitt, André M. Toschke, Andy P. Grieve, Anthony G. Rudd

**Affiliations:** 1 Division of Health and Social Care Research, King's College London, London, United Kingdom; 2 National Institute for Health Research Comprehensive Biomedical Research Centre, Guy's and St Thomas' NHS Foundation Trust and King's College London, London, United Kingdom; 3 Centre for Stroke Research Berlin, Charite—Universitaetsmedizin Berlin, Berlin, Germany; 4 Department of Medical Informatics, Biometry and Epidemiology, University of Munich, Munich, Germany; University of Edinburgh, United Kingdom

## Abstract

**Background:**

Although stroke is acknowledged as a long-term condition, population estimates of outcomes longer term are lacking. Such estimates would be useful for planning health services and developing research that might ultimately improve outcomes. This burden of disease study provides population-based estimates of outcomes with a focus on disability, cognition, and psychological outcomes up to 10 y after initial stroke event in a multi-ethnic European population.

**Methods and Findings:**

Data were collected from the population-based South London Stroke Register, a prospective population-based register documenting all first in a lifetime strokes since 1 January 1995 in a multi-ethnic inner city population. The outcomes assessed are reported as estimates of need and included disability (Barthel Index <15), inactivity (Frenchay Activities Index <15), cognitive impairment (Abbreviated Mental Test < 8 or Mini-Mental State Exam <24), anxiety and depression (Hospital Anxiety and Depression Scale >10), and mental and physical domain scores of the Medical Outcomes Study 12-item short form (SF-12) health survey. Estimates were stratified by age, gender, and ethnicity, and age-adjusted using the standard European population. Plots of outcome estimates over time were constructed to examine temporal trends and sociodemographic differences. Between 1995 and 2006, 3,373 first-ever strokes were registered: 20%–30% of survivors had a poor outcome over 10 y of follow-up. The highest rate of disability was observed 7 d after stroke and remained at around 110 per 1,000 stroke survivors from 3 mo to 10 y. Rates of inactivity and cognitive impairment both declined up to 1 y (280/1,000 and 180/1,000 survivors, respectively); thereafter rates of inactivity remained stable till year eight, then increased, whereas rates of cognitive impairment fluctuated till year eight, then increased. Anxiety and depression showed some fluctuation over time, with a rate of 350 and 310 per 1,000 stroke survivors, respectively. SF-12 scores showed little variation from 3 mo to 10 y after stroke. Inactivity was higher in males at all time points, and in white compared to black stroke survivors, although black survivors reported better outcomes in the SF-12 physical domain. No other major differences were observed by gender or ethnicity. Increased age was associated with higher rates of disability, inactivity, and cognitive impairment.

**Conclusions:**

Between 20% and 30% of stroke survivors have a poor range of outcomes up to 10 y after stroke. Such epidemiological data demonstrate the sociodemographic groups that are most affected longer term and should be used to develop longer term management strategies that reduce the significant poor outcomes of this group, for whom effective interventions are currently elusive.

*Please see later in the article for the Editors' Summary*

## Abstract 2

Charles Wolfe and colleagues collected data from the South London Stroke Register on 3,373 first strokes registered between 1995 and 2006 and showed that between 20% and 30% of survivors have poor outcomes up to 10 years after stroke.

## Editors' Summary

**Background:**

Every year, 15 million people have a stroke. About 5 million of these people die within a few days, and another 5 million are left disabled. Stroke occurs when the brain's blood supply is suddenly interrupted by a blood clot blocking a blood vessel in the brain (ischemic stroke, the commonest type of stroke) or by a blood vessel in the brain bursting (hemorrhagic stroke). Deprived of the oxygen normally carried to them by the blood, the brain cells near the blockage die. The symptoms of stroke depend on which part of the brain is damaged but include sudden weakness or paralysis along one side of the body, vision loss in one or both eyes, and confusion or trouble speaking or understanding speech. Anyone experiencing these symptoms should seek immediate medical attention because prompt treatment can limit the damage to the brain. Risk factors for stroke include age (three-quarters of strokes occur in people over 65 years old), high blood pressure, and heart disease.

**Why Was This Study Done?:**

Post-stroke rehabilitation can help individuals overcome the physical disabilities caused by stroke, and drugs and behavioral counseling can reduce the risk of a second stroke. However, people can also have problems with cognition (thinking, awareness, attention, learning, judgment, and memory) after a stroke, and they can become depressed or anxious. These “outcomes” can persist for many years, but although stroke is acknowledged as a long-term condition, most existing data on stroke outcomes are limited to a year after the stroke and often focus on disability alone. Longer term, more extensive information is needed to help plan services and to help develop research to improve outcomes. In this burden of disease analysis, the researchers use follow-up data collected by the prospective South London Stroke Register (SLSR) to provide long-term population-based estimates of disability, cognition, and psychological outcomes after a first stroke. The SLSR has recorded and followed all patients of all ages in an inner area of South London after their first-ever stroke since 1995.

**What Did the Researchers Do and Find?:**

Between 1995 and 2006, the SLSR recorded 3,373 first-ever strokes. Patients were examined within 48 hours of referral to SLSR, their stroke diagnosis was verified, and their sociodemographic characteristics (including age, gender, and ethnic origin) were recorded. Study nurses and fieldworkers then assessed the patients at three months and annually after the stroke for disability (using the Barthel Index, which measures the ability to, for example, eat unaided), inactivity (using the Frenchay Activities Index, which measures participation in social activities), and cognitive impairment (using the Abbreviated Mental Test or the Mini-Mental State Exam). Anxiety and depression and the patients' perceptions of their mental and physical capabilities were also assessed. Using preset cut-offs for each outcome, 20%–30% of stroke survivors had a poor outcome over ten years of follow-up. So, for example, 110 individuals per 1,000 population were judged disabled from three months to ten years, rates of inactivity remained constant from year one to year eight, at 280 affected individuals per 1,000 survivors, and rates of anxiety and depression fluctuated over time but affected about a third of the population. Notably, levels of inactivity were higher among men than women at all time points and were higher in white than in black stroke survivors. Finally, increased age was associated with higher rates of disability, inactivity, and cognitive impairment.

**What Do These Findings Mean?:**

Although the accuracy of these findings may be affected by the loss of some patients to follow-up, these population-based estimates of outcome measures for survivors of a first-ever stroke for up to ten years after the event provide concrete evidence that stroke is a lifelong condition with ongoing poor outcomes. They also identify the sociodemographic groups of patients that are most affected in the longer term. Importantly, most of the measured outcomes remain relatively constant (and worse than outcomes in an age-matched non-stroke-affected population) after 3–12 months, a result that needs to be considered when planning services for stroke survivors. In other words, these findings highlight the need for health and social services to provide long-term, ongoing assessment and rehabilitation for patients for many years after a stroke.

**Additional Information:**

Please access these Web sites via the online version of this summary at http://dx.doi.org/10.1371/journal.pmed.1001033.

- The US National Institute of Neurological Disorders and Stroke provides information about all aspects of stroke (in English and Spanish); the US National Institute of Health SeniorHealth Web site has additional information about stroke
- The Internet Stroke Center provides detailed information about stroke for patients, families, and health professionals (in English and Spanish)
- The UK National Health Service Choices Web site also provides information about stroke for patients and their families
- MedlinePlus has links to additional resources about stroke (in English and Spanish)
- More information about the South London Stroke Register is available

## Introduction

The World Health Organization's Global Burden of Disease analyses rely on routine mortality and limited disability data throughout most countries worldwide. These data have persistently highlighted stroke as the fourth leading cause of disability-adjusted life years (DALYs) lost (stroke accounts for 6.3% DALYs, equating to 83.61 million DALYs in low and middle income countries and 9.35 million DALYs in high income countries) [1]. To estimate DALYs, a range of data sources, including disease registers, epidemiological studies, and health surveys, are utilised, yet the data that inform the DALY estimates for long-term planning are not at all comprehensive.

Stroke is a condition that requires long-term management, and some strategies to address such issues as rehabilitation, psychological treatments, and social support have been advocated at a national level in the United Kingdom [2]. Yet estimates of different outcomes after stroke in the long term, after 1 y, are lacking, with most of the existing data on stroke outcomes and costs being restricted to short-term cohort studies with limited follow-up (usually up to 1 y), as well as focussing on disability alone or relatively few outcome measures only. Additionally, selection bias due to inclusion of only patients referred to hospitals and/or rehabilitation settings often occurs. In the few population-based follow-up studies, quality of life has been assessed between 2 and 21 y after stroke [3]–[7], and activities of daily living have been assessed at 1, 3, 8, 16, and 21 y after stroke in a follow-up study in Auckland [3], up to 5 y after stroke in Perth, Australia, and 5 y after stroke in South London [7]–[9].

The aim of this burden of disease study is to generate population-based estimates of long-term outcomes after stroke using data for up to 10 y of follow-up in an unbiased population sample, the South London Stroke Register (SLSR).

## Methods

### Study Population

The SLSR is a prospective population-based stroke register set up in January 1995, recording all first-ever strokes in patients of all ages for an inner area of South London based on 22 electoral wards in Lambeth and Southwark. Data collected between 1995 and 2006 were used in this analysis, and the denominator population was derived from 1991 and 2001 Census data with mid-year adjustments [10],[11].

The total source population of the SLSR area was 271,817 individuals, self-reported as 63% White, 28% Black (9% Black Caribbean, 15% Black African, and 4% Black Other), and 9% Of Other Ethnic Group in the 2001 census. Between the most recent censuses of 1991 and 2001, the proportion of individuals in ethnic groups other than White increased from 28% to 37%; in 1991, the largest ethnic minority group was Black Caribbeans (11%), but by 2001, Black Africans made up the largest ethnic minority group (15%) [10],[11].

### Case Ascertainment

Standardised criteria were applied to ensure completeness of case ascertainment, including multiple overlapping sources of notification [10],[11]. Stroke was defined according to World Health Organization criteria [10], and all subarachnoid haemorrhages (ICD-10 code I60.–), intracerebral haemorrhages (I61.–), cerebral infarctions (I63.–), and unspecified strokes (I64) were included. Patients admitted to hospitals serving the study area (two teaching hospitals within and three hospitals outside the study area) were identified by regular reviews of acute wards admitting stroke patients, weekly checks of brain imaging referrals, and monthly reviews of bereavement officer and bed manager records. Additionally, national data on patients admitted to any hospital in England and Wales with a diagnosis of stroke were also screened for additional patients. To identify patients not admitted to hospital, all general practitioners within and on the borders of the study area were contacted regularly and asked to notify the SLSR of stroke patients. Regular communication with general practitioners was achieved by telephone contact and quarterly newsletters. Referral of non-hospitalised stroke patients to a neurovascular outpatient clinic (from 2003) or domiciliary visit to patients by the study team was also available to general practitioners. Community therapists were contacted every 3 mo. Death certificates were checked regularly. Completeness of case ascertainment has been estimated at 88% by a multinomial-logit capture-recapture model using the methods described in detail elsewhere [10].

### Data Collection

Specially trained study nurses and field workers collected all data prospectively whenever feasible. A study doctor verified the diagnosis of stroke. Patients were examined within 48 h of referral to SLSR where possible. The following sociodemographic characteristics were collected at initial assessment: self-definition of ethnic origin (census question), stratified into White, Black (Black Caribbean, Black African, and Black Other), and Other Ethnic Group. Socioeconomic status was categorised as non-manual (I, II, and III non-manual), manual (III manual, IV, and V), and economically inactive (retired and no information on previous employment), according to the patient's current or most recent employment using the UK General Register Office occupational codes. Classification of pathological stroke subtype (ischaemic stroke, primary intracerebral haemorrhage, or subarachnoid haemorrhage) was based on results from at least one of the following: brain imaging performed within 30 d of stroke onset (computerised tomography or magnetic resonance imaging), cerebrospinal fluid analysis (in all living cases of subarachnoid haemorrhage where brain imaging was not diagnostic), or necropsy examination. Cases without pathological confirmation of stroke subtype were classified as undefined [10],[11]. The Glasgow Coma Score dichotomised to <13 or ≥13 was used as a standardised measure of stroke severity (Table 1) [10],[11].

**Table 1:** Sociodemographics, stroke subtype, and case fatality of SLSR patients, 1995–2006.

Characteristic	Subcategory	Value (*n* = 3,373)
**Age, mean (standard deviation)**		70.3 (14.6)
**Age, ** ***n*** ** (%)**	<65 y	1,038 (30.8)
	65–74 y	891 (26.4)
	75–84 y	956 (28.3)
	85+y	488 (4.5)
**Female sex, ** ***n*** ** (%)**		1,663 (49.3)
**Ethnicity, ** ***n*** ** (%)**	White	2,451 (72.7)
	Black	645 (19.1)
	Other	187 (5.5)
	Unknown	90 (2.7)
**Socioeconomic status, ** ***n*** ** (%)**	Non-manual	870 (26.7)
	Manual	1,877 (55.3)
	Economically inactive	499 (14.8)
	Unknown	127 (3.8)
**BI prior to stroke, ** ***n*** ** (%)**	20, independent	2,505 (77.8)
	15–19, mild disability	492 (15.3)
	0–14, moderate-severe disability	225 (7.0)
**Stroke subtype, ** ***n*** ** (%)**	Infarction	2,470 (76.5)
	Primary intracerebral haemorrhage	464 (13.8)
	Subarachnoid haemorrhage	193 (5.7)
	Undefined	246 (7.3)
**Cumulative survival, % (95% CI)**	1 y	63.7 (61.2–65.3)
	5 y	42.8 (41.0–44.5)
	10 y	24.0 (22.1–26.0)

Follow-up data were collected by validated postal or face-to-face instruments with patients and/or their carers, the interview lasting less than 1 h. If a patient had left the SLSR area, they were followed up if at all possible using the methods described. Patients were assessed at 3 mo and annually after stroke. All follow-up assessments included in the present study were completed by 31 August 2009. Outcome measures included activity of daily living using the Barthel Index (BI) [12], extended activities of daily living (social activities) using the Frenchay Activities Index (FAI) [13], health-related quality of life (HRQOL) using the UK version of the Medical Outcomes Study 12-item short form (SF-12) or 36-item short form (SF-36) surveys [14],[15], cognitive impairment using the Mini-Mental State Exam (MMSE) [16] or Abbreviated Metal Test [17], and anxiety and depression using the Hospital Anxiety and Depression Scale [18]. All interviewers underwent regular standardised training in the use of the different scales.

Cut-off points for determining poor outcomes were defined a priori. The BI was assessed in the acute phase (7–10 d after stroke) and at all follow-up interviews. A score on the BI of <15 was used to identify patients with moderate (BI = 10–14) to severe (BI <9) disability [19]. The FAI was administered at all follow-up points, and participants with a score <15 categorised as “inactive” [20].

The SF-36 was used to measure HRQOL in follow-up interviews conducted before 1 March 1999, after which the shortened version, the SF-12, was introduced. The 12 items of the SF-12 have been adopted from the SF-36 verbatim, and summary scores are replicable and reproducible [15],[21]. Therefore, the specific items from the SF-36 questionnaires in earlier follow-ups were used to derive SF-12 summary scores across all time points. The SF-12 was selected to measure HRQOL because of its strong psychometric properties, wide use, reliability, validity, and responsiveness [20],[22]. It assesses eight domains of health status, called physical functioning, role physical, bodily pain, general health, vitality, social functioning, role emotional, and mental health. Each domain is scored from 0 to 100. Absence of problems is indicated by scores of 100 for physical functioning, role physical, bodily pain, social functioning, and role emotional, and scores of 50 in general health, vitality, and mental health. These domains were then used to produce two summary scores representing physical and mental health [15]. Domains for the physical health summary score included physical functioning, role physical, bodily pain, and general health. The mental health summary score included the domains vitality, social functioning, role emotional, and mental health. The summary scores ranged from 0 to 100 and were based on norms with a mean of 50 and a standard deviation of 10. Summary scores in this study are presented as 100-score, with higher values signifying poorer outcome.

Cognitive state was assessed in the acute phase as well as at follow-up. Prior to 1 January 2000, all assessments were conducted using the MMSE; after 1 January 2000, the Abbreviated Metal Test was administered. Subjects were defined as cognitively impaired according to predefined cut-off points (MMSE <24 or Abbreviated Mental Test <8) [22],[23].

The Hospital Anxiety and Depression Scale, consisting of two subscales, was originally developed as a screening tool for anxiety and depression in hospital patients but has also been validated for use in stroke patients [24] and in the general population [25]. Each subscale is scored from 0 to 21 and used to identify possible (score >7) cases of anxiety and depression [25].

Table 2 details the benchmarking of outcomes with non-stroke population samples. We searched for papers with outcomes identical to those of this study and with age groups as near as possible to those of this study. Apart from the PubMed search we also included data from the Health Survey for England [26]–[31].

**Table 2:** Population estimates of outcomes measured in the SLSR follow-up assessments.

Measure	SLSR Estimate for Stroke Patients	Non-Stroke Population Estimate	Reference for Non-Stroke Population Estimate
Disability	11%	37% men; 40% women at least one functional limitation (>65 y)	26
Cognition	18% (MMSE <24)	>65 y: 8.5%–9.8%, >75 y: 18.3% (MMSE <22)	27
		75–79 y: 11.2% ≥80 y: 46.5% (MMSE <24)	28
		>65 y: 4.6% (CARE Schedule^a^)	29
Depression	31%	8.7%–13.5%	29,30
Anxiety	35%	3.7%	29
SF-12 physical health, age <65 y	62.3	50.0	31
SF-12 physical health, age 65–74 y	64.2	54.7	31
SF-12 physical health, age ≥75 y	65.4		
SF-12 mental health, age <65 y	54.7	48.6	31
SF-12 mental health, age 65–74 y	52.1	46.8	31
SF-12 mental health, age ≥75 y	51.8		

For SF-12 scores, higher score indicates poorer health.

a A validated structured interview schedule that includes an “organic brain syndrome” subscale, used to identify cognitive impairment.

### Statistical Analysis

Kaplan–Meier estimates were used to model survival and to measure the cumulative survival and 95% confidence intervals at 1, 5, and 10 y after stroke. Proportions and pointwise 95% confidence intervals were calculated based on the binomial distribution at all time points for rates of disability, inactivity (extended activities of daily living), cognitive impairment, anxiety, and depression [32]. For the SF-12 mental and physical domains, means and pointwise 95% confidence intervals were calculated using the Student's *t*-distribution. Estimates were stratified by gender, age, and ethnicity. The standard European population [33] was used to provide age-adjusted estimates in all analyses apart from those stratified by age. All data available at each time point were considered.

A number of sensitivity analyses were carried out to assess the robustness of results. Possible changes in outcomes by calendar year were assessed by analysing rates and means at 1 and 5 y after stroke by year of stroke. In a complete case analysis, only survivors with data at all points up to 5 y after stroke were considered. In a final analysis, missing data for survivors were imputed at all time points using a best- and then worst-case scenario for binary outcomes and assuming a score of 50 in the SF-12 domains.

**Figure 1:**
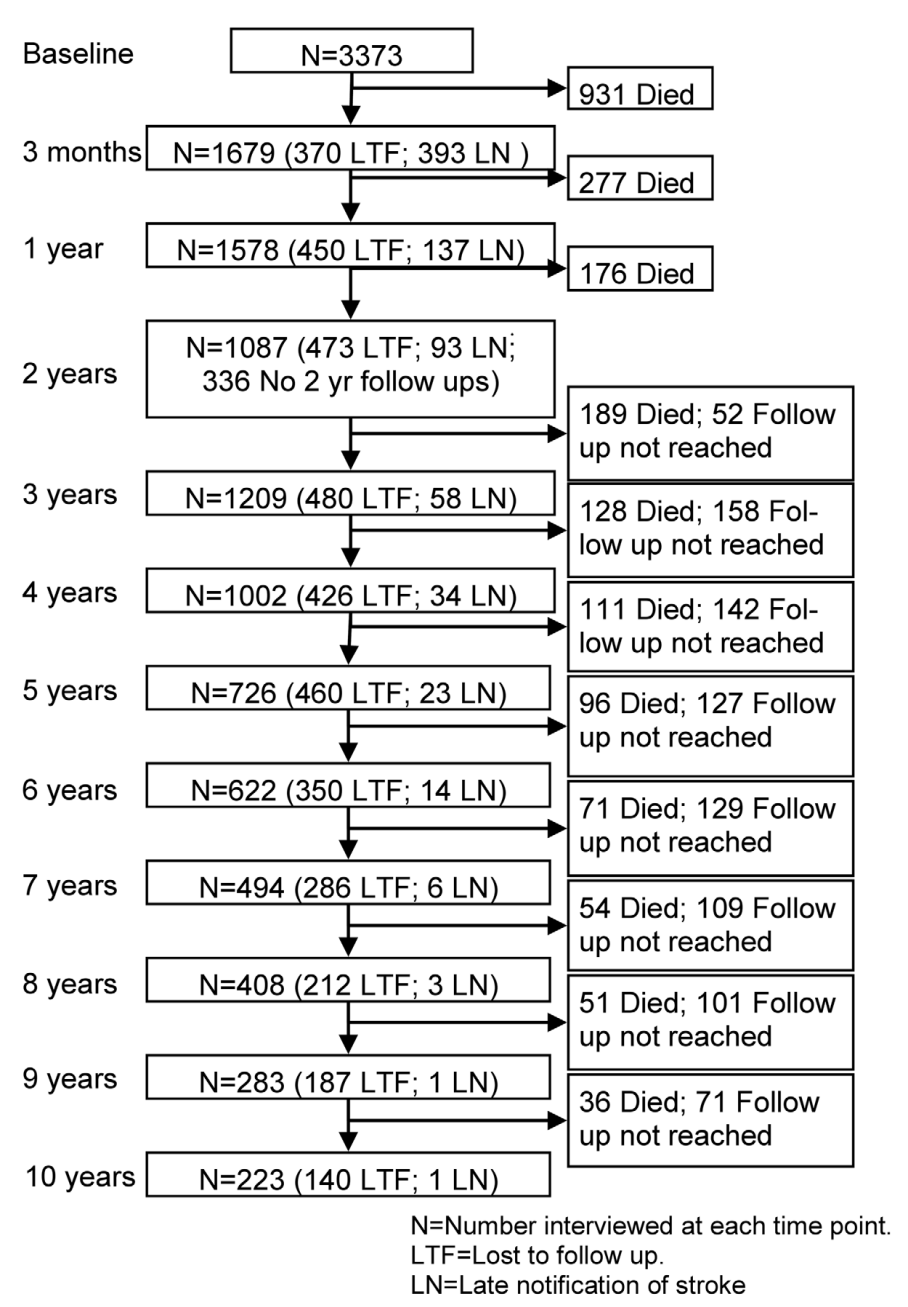
Flow chart showing the number of participants included at each follow-up time point.

Loss to follow-up rates varied by time point (after accounting for deaths): 3 mo (24%); 1 y (17.9%); 2 y (29.1%, but data not collected in 1998/1999); 3 y (18.9%); 4 y (16.8%); 5 y (18.5%); 6 y (15.4%); 7 y (14.2%); 8 y (12.3%), 9 y (12.6%); 10 y (11.7%). Figure 1 details the follow-up annually of this cohort over the 10 y. The number of patients who died between two time points and the number not eligible due to the later time point not yet being reached are provided in the right-hand column. These participants are subsequently ineligible for any future follow-up. In the left-hand column the numbers followed up are included, with details of those lost to follow-up and notified late. Late notification refers to those not notified until after the specified time point in the Figure 1; for example, lost notification at 9 y was in a patient first identified at 9 y after the initial event.

These participants (lost to follow-up and late notifications) remain in the sample eligible for future follow-ups. All analyses were performed using Stata 10SE [34] and R 2.8.1 [35].

### Ethics

All patients and/or their relatives gave written informed consent to participate in the study, and over the study period very few patients have declined to be registered. The design of the study was approved by the ethics committees of Guy's and St Thomas' NHS Foundation Trust, King's College Hospital Foundation Trust, St George's University Hospital, National Hospital for Nervous Diseases, and Westminster Hospital.

## Results

A total of 3,373 patients with first-ever stroke between 1 January 1995 and 31 December 2006 were registered in the SLSR. The sociodemographic data, pathological stroke subtype data, and case fatality rates are presented in Table 1. Mean age was 70.3 y (standard deviation 14.6), and 49.3% were female (Table 1). Most patients were white (72.7%), followed by black (Black African and Black Caribbean) (19.1%), while other or unknown ethnicity was recorded in less than 10%. The majority of patients were classified as independent by the BI prior to stroke (77.8%). Ischaemic strokes were observed in 76.5%, primary intracerebral haemorrhage in 13.8%, and subarachnoid haemorrhage in 5.7%. The Glasgow Coma Score dichotomised to <13 or ≥13, as a standardised measure of stroke severity, showed no change over time after adjusting for age, gender, ethnicity, and subtype of stroke.

**Figure 2:**
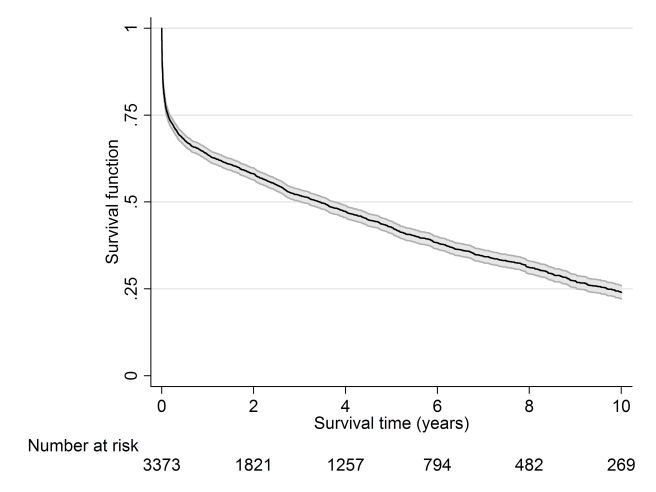
Kaplan–Meier survival estimates with 95% confidence intervals.

Cumulative survival up to 10 y after stroke is displayed in Figure 2, with 63.7%, 42.8%, and 24.0% surviving up to 1, 5, and 10 y, respectively.

**Figure 3:**
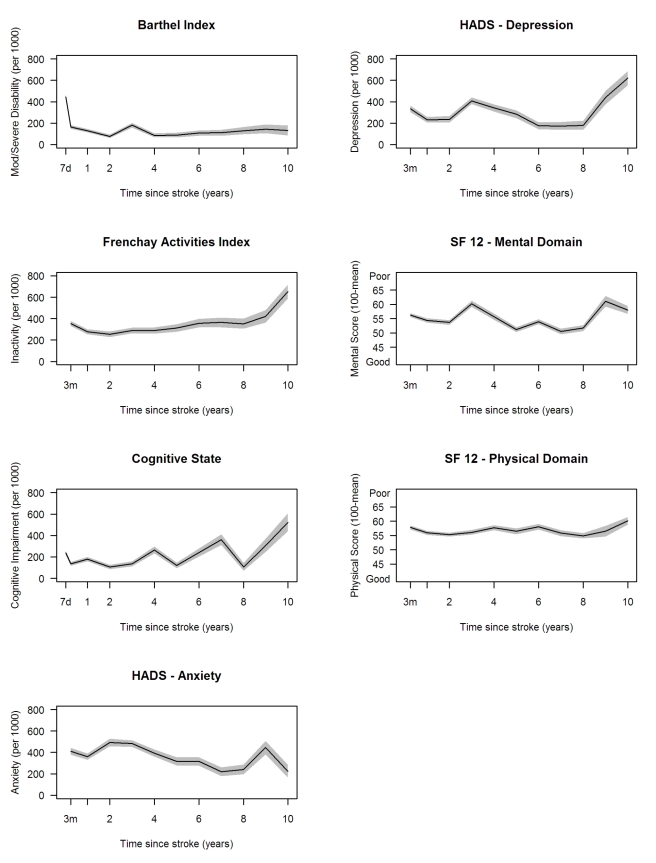
Age-adjusted rates of outcome per 1,000 stroke suvivors, with 95% pointwise confidence intervals. HADS, Hospital Anxiety and Depression Scale.

The highest proportion of disabled stroke survivors was observed 7 d after stroke, while the proportion remained at around 110 per 1,000 stroke survivors after 3 mo (Figure 3).

Rates of inactivity, measured by the FAI, declined in the first year after stroke, then remained stable till year eight, then increased, whereas rates of cognitive impairment fluctuated till year eight, then increased. Anxiety and depression showed variation up to 10 y, with average rates around 350 and 310 per 1,000 population, respectively. Mean HRQOL physical domain stroke summary scores were also quite stable from 3 mo to 10 y after stroke (Figure 3), whereas mental domain stroke summary scores fluctuated.

**Figure 4:**
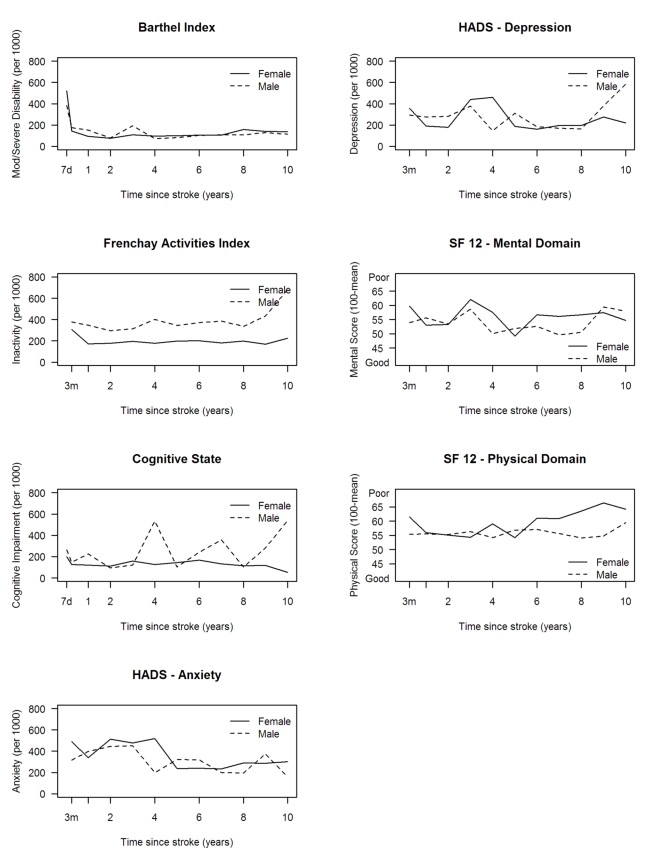
Age-adjusted rates of outcome per 1,000 stroke suvivors by gender. HADS, Hospital Anxiety and Depression Scale.

**Figure 5:**
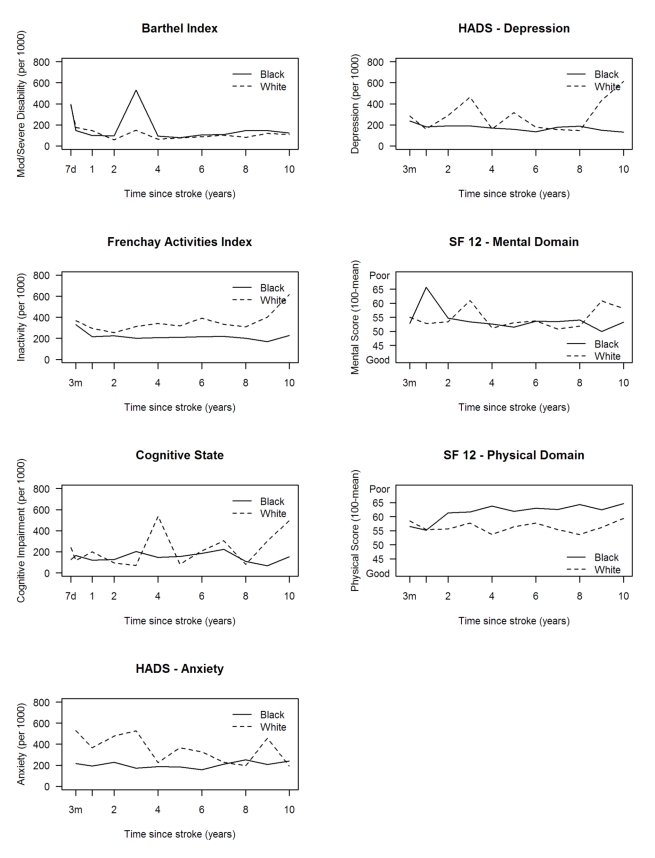
Age-adjusted rates of outcome per 1,000 stroke suvivors and mean SF-12 scores by ethnicity. HADS, Hospital Anxiety and Depression Scale.

**Figure 6:**
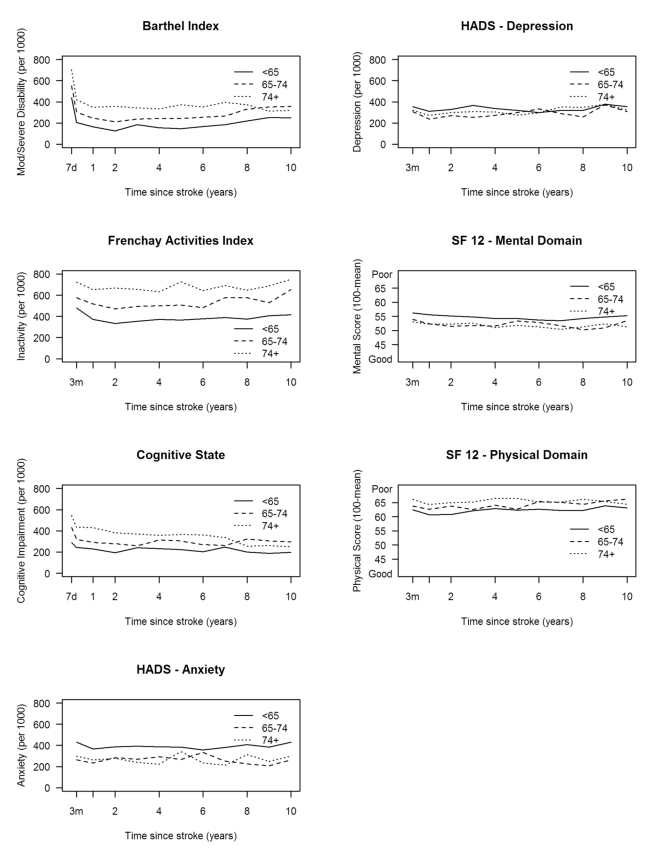
Rates of outcome per 1,000 stroke suvivors and mean SF-12 scores by age. HADS, Hospital Anxiety and Depression Scale.

Levels of inactivity (FAI) were higher in males at all time points (Figure 4). No other major differences were observed between males and females. Higher levels of inactivity (FAI) were observed in white compared with black stroke survivors, although the white group showed a more favourable outcome in the HRQOL physical domain (Figure 5). Age was directly associated with rates of disability, inactivity, and cognitive impairment, while there was no clear association between age and anxiety and depression and SF-12 mental and physical domains (Figure 6).

In sensitivity analyses, the rates and means of all outcomes at 1 and 5 y after stroke did not show large variation by year of stroke (Figure S1). Additionally, complete case analysis showed rates and means similar to those of the original analysis over the first 5 y of follow-up (Figure S2). When best- and worst-case imputation methods were applied, although overall rates were altered, the trends over time closely followed those in the observed and complete case analyses (Figure S3).

## Discussion

This study analyses a population-based cohort of stroke patients followed for up to 10 y. It not only provides population estimates, to our knowledge for the first time, on the longer term outcomes in a diverse inner city population but highlights that stroke is truly a lifelong condition among survivors with ongoing poor outcomes. A major observation is that after 3–12 mo the outcomes remain relatively constant. There are some differences in the rates of the different outcomes between sociodemographic groups that are largely unexplained, but the effect of age on poorer outcomes indicates a challenge to be faced in future years [36].

It is rare that population-based studies estimate this range of outcomes in such a prospective manner, with up to 10 y of follow-up. Previous studies have addressed very long term outcomes, but only for certain selected outcomes and not annually [3]–[8]. The use of these year-on-year point prevalence estimates, in, for example, the World Health Organization's Global Burden of Disease estimates of DALYs, would provide more precise estimates based on population observations [8].

This burden of disease study only estimates outcomes in stroke survivors, with no comparison to non-stroke populations. The data have not been analysed with prediction of outcome as a focus, and further analyses of patterns and predictors of outcome in various sociodemographic, stroke subtype, and case mix groups are required to develop clinically useful prediction tools. For example, in the early assessment time points, patients with severe stroke are included, and the rates of poor outcome might intuitively be thought to be higher, but as individuals in this group die and patients who had milder strokes survive, rates of poor outcome may be expected to reduce. Another factor that may influence the estimates of outcome and determine differences between groups is stroke care itself, although the year of stroke in this analysis had no effect on patterns of outcome. Previous work by McKevitt et al. [37] did not find that any specific sociodemographic factors influenced the uptake of effective acute stroke care and early secondary prevention interventions in this population [37].

Table 2 benchmarks the outcome estimates from this study with age-matched UK population survey data where the same or very similar outcome instruments have been employed, and although such comparisons are not as ideal as a case-control design to estimate outcome differences, they do largely indicate poorer outcomes in the stroke population, re-enforcing the World Health Organization's Global Burden of Disease analyses, except for disability, where no population norms were reported using the BI or a similar scale [1].

Disability has been reported up to 5 y after stroke, and a delayed but significant functional decline has been observed in survivors [38]. In this study, there was, as anticipated, a dramatic reduction in activities of daily living to 2 y, followed by an improvement and then a plateau, but with 10%–20% of patients having moderate to severe disability at 10 y. Although the evidence base for rehabilitation interventions early after stroke is strong, how to reduce stroke-related disability in the longer term remains unclear. Yet these estimates highlight that 20%–30% of patients at any time point presumably require some sort of ongoing assessment and rehabilitation intervention.

Activity, as measured by the FAI, remains relatively stable over time, but with around 30% of survivors being classified as inactive. There is an increase in inactivity, after adjustment for age, after 8 y, which may be a result of residual confounding from other comorbidities. Activity may well be linked to disability but will also have other drivers, and assessment of patients in terms of mobility and ability to integrate into society should be canvassed and solutions found either at a patient or group level.

We have previously reported that up to 3 y after stroke cognitive impairment is present in approximately one-third of survivors assessed using the MMSE [39]. Rates of cognitive deficit fluctuate in this cohort to 8 y, then increase, and this may represent progressive vascular dementia associated with stroke, although we did not observe any particular patterns with age.

In a systematic review of the literature on post-stroke depression, Hackett et al. [40] highlighted the range of different scales and cut-offs used to define depression. The pooled estimate of all stroke survivors experiencing depression was 33%, although the maximum follow-up in these studies was 3 y [40]. Data from our analyses confirm fluctuation in rates of depression over 10 y, with an average of 31% of patients having depression. In Martinique, depression at 5 y after stroke was estimated at 25.8%, using the Montgomery-Asberg Depression Rating Scale [41].

HRQOL has been assessed up to 21 y after stroke in New Zealand [2],[3]. At 6 y after stroke, HRQOL was found to be “acceptable” for the majority of survivors, even though many experienced ongoing limitation of physical function. At 21 y after stroke, standardised mean SF-36 scores were similar to those for the age-matched non-stroke population, suggesting that stroke survivors live relatively successfully within the general population, despite ongoing disability [3]. In this study, HRQOL scores fluctuated around 50–60, with 100 representing poor HRQOL scores in both physical and mental domains, and further analyses of the relationship between HRQOL and the other domains of outcome are required to fully understand why, in the face of significant loss of activity and participation, HRQOL for stroke patients appears to compare favourably with non-stroke population values. There are unexplained fluctuations in the mental domain estimates over time that are not observed in physical outcomes.

The loss to follow-up rates, once deaths are accounted for, in this study are less than 20% at each time point except at 3 mo and 2 y. One might have expected the highest follow-up rate at 3 mo; however, a proportion of patients are registered retrospectively for whom 3-mo assessment is not possible. This loss to follow-up may introduce bias, yet estimates from analyses of the patients with complete data did not differ significantly from those presented here. Loss to follow-up may be an issue in certain sociodemographic groups, although we have not been able to identify such groups in this analysis. The healthier participants and those from higher socioeconomic groups may be more likely to engage in research follow-up. In other cohort and stroke register studies, loss to follow-up rates are not often presented. Inner city populations are mobile, with large numbers of migrant families. Although we acknowledge this as a potential factor in loss to follow-up, efforts were made for all patients' changes of address to be recorded from either hospital, general practice, or family sources. Patients and their families were then assessed face to face if at all possible, but if they had moved to another country, postal questionnaires were often sent and returned.

This population-based study has produced estimates of outcome clearly demonstrating the long-term nature of disabilities following stroke. Such estimates can be incorporated into estimated DALYs for stroke and serve as objective estimates of need for stroke patients. These estimates should highlight to health and social service providers that stroke patients should not be lost to the health and social care system and that providers will need to develop innovative solutions to address the poor outcomes after stroke in the long term.

## Supporting Information

Figure S1 **Observed rates of outcomes at 1 and 5** **y after stroke by year of stroke.**
(TIFF)

Figure S2 **Observed age-adjusted rates of outcomes and estimated rates using imputation in survivors who were lost to follow-up.**
(TIFF)

Figure S3 **Age-adjusted rates of outcomes per 1,000 survivors with complete data up to 5** **y after stroke.**
(TIFF)

## Abbreviations

BI: Barthel Index
DALY: disability-adjusted life year
FAI: Frenchay Activities Index
HLQOL: health-related quality of life
MMSE: Mini-Mental State Exam
SF-12: Medical Outcomes Study 12-item short form
SF-36: Medical Outcomes Study 36-item short form
SLSR: South London Stroke Register

## References

[1] Lopez AD, Mathers CD, Ezzati M, Jamison DT, Murray CJL (2006). Global and regional burden of disease and risk factors, 2001:
systematic analysis of population health data.. Lancet.

[2] Department of Health (2007). National stroke strategy.. http://www.dh.gov.uk/en/Publicationsandstatistics/Publications/PublicationsPolicyandGuidance/DH_081062.

[3] Anderson CS, Carter KN, Brownlee WJ, Hackett ML, Broad JB (2004). Very long-term outcome after stroke in Auckland, New
Zealand.. Stroke.

[4] Hackett ML, Duncan JR, Anderson CS, Broad JB, Bonita R (2000). Health-related quality of life among long-term survivors of
stroke: results from the Auckland Stroke Study, 1991-1992.. Stroke.

[5] Niemi ML, Laaksonen R, Kotila M, Waltimo O (1988). Quality of life 4 years after stroke.. Stroke.

[6] Paul SL, Sturm JW, Dewey HM, Donnan GA, Macdonell RA (2005). Long-term outcome in the North East Melbourne Stroke Incidence
Study: predictors of quality of life at 5 years after
stroke.. Stroke.

[7] Hankey GJ, Jamrozik K, Broadhurst RJ, Forbes S, Anderson CS (2002). Long-term disability after first ever stroke and related
prognostic factors in the Perth community stroke study,
1989-1990.. Stroke.

[8] Patel MD, Tilling K, Lawrence E, Rudd AG, Wolfe CDA (2007). Relationships between long-term stroke disability, handicap and
health-related quality of life.. Age Ageing.

[9] Wilkinson PR, Wolfe CDA, Warburton FG, Rudd AG, Howard RS (1997). A long-term follow-up of stroke patients.. Stroke.

[10] Heuschmann PU, Grieve AP, Toschke AM, Rudd AG, Wolfe CDA (2008). Ethnic group disparities in 10-year trends in stroke incidence
and vascular risk factors: The South London Stroke Register
(SLSR).. Stroke.

[11] Wolfe CD, Rudd AG, Howard R, Coshall C, Stewart J (2002). Incidence and case fatality rates of stroke subtypes in a
multiethnic population: the South London Stroke Register.. J Neurol Neurosurg Psychiatry.

[12] Wade DT, Collin C (1988). The Barthel ADL Index: a standard measure of physical
disability?. Int Disabil Stud.

[13] Wade DT, Legh-Smith J, Langton Hewer J (1985). Social activities after stroke: measurement and natural history
using Frenchay Activities Index.. Int Rehabil Med.

[14] Ware JE, Kosinski M, Keller SD (1994). SF-36 physical and mental health summary scales: a user's
manual..

[15] Ware JE, Kosinski M, Keller SD (1998). SF-12: How to score the SF-12 physical and mental health summary
scales, 3^rd^ edition..

[16] Folstein M, Folstein S, McHugh P (1975). “Mini-mental state”: A practical method of grading
the cognitive state of patients for the clinician.. J Psychiatr Res.

[17] Hodkinson H (1972). Evaluation of a mental test score for assessment of mental
impairment in the elderly.. Age Ageing.

[18] Zigmond AS, Snaith RP (1983). The hospital anxiety and depression scale.. Acta Psychiatr Scand.

[19] Wolfe CDA, Taub NA, Woodrow J, Burney PGJ (1991). Assessment of scales of disability and handicap for stroke
patients.. Stroke.

[20] Anderson CS, Jamrozik KD, Broadhurst RJ, Stewart-Wynne EG (1994). Predicting survival for 1 year among different subtypes of
stroke.. Stroke.

[21] Pickard AS, Johnson JA, Penn A, Lau F, Noseworthy T (1999). Replicability of SF-36 summary scores by the SF-12 in stroke
patients.. Stroke.

[22] Tombaugh TN, McIntyre NJ (1992). The mini-mental state examination: a comprehensive
review.. J Am Geriatr Soc.

[23] Jitapunkul S, Pillay I, Ebrahim S (1991). The abbreviated mental test: its use and
validity.. Age Ageing.

[24] Aben I, Verhey F, Lousberg R, Lodder J, Honig A (2002). Validity of the Beck Depression Inventory, Hospital Anxiety and
Depression Scale, SCL-90, and Hamilton Depression Rating Scale as screening
instruments for depression in stroke patients.. Psychosomatics.

[25] Bjelland I, Dahl A, Haug TT, Neckelmann D (2002). The validity of the Hospital Anxiety and Depression Scale. An
updated literature review.. J Psychosom Res.

[26] Craig R, Mindell J (2005). Health survey for England 2005: health of older
people.. Volume 1, General health and function.

[27] The MedicAgeing Study (1998). Cognitive function and dementia in six areas of England and
Wales: the distribution of MMSE and prevalence of GMS organicity level in
the MRC CFA Study.. Psychol Med.

[28] Rait G, Fletcher A, Smeeth L, Brayne C, Stirling S (2005). Prevalence of cognitive impairment: results from the MRC trial of
assessment and management of older people in the community.. Age Ageing.

[29] Lindesay J, Briggs K, Murphy E (1989). The Guy's/Age Concern survey. Prevalence rates of cognitive
impairment, depression and anxiety in an urban elderly
community.. Br J Psychiatry.

[30] McDougall FA, Kvaal K, Matthews FE, Paykel E, Jones PB (2007). Prevalence of depression in older people in England and Wales:
the MRC CFA Study.. Psychol Med.

[31] Gandek B, Ware JE, Aaronson NK, Apolone G, Bjorner JB (1998). Cross-validation of item selection and scoring for the SF-12
Health Survey in nine countries: results from the IQOLA Project.
International Quality of Life Assessment.. J Clin Epidemiol.

[32] Clopper C, Pearson S (1934). The use of confidence or fiducial limits illustrated in the case
of the binomial.. Biometrika.

[33] Sharp L, Black RJ, Harkness EF, Finlayson AR, Muir CS (1993). Cancer registration statistics, Scotland
1981–1990..

[34] StataCorp (2007). Stata statistical software: release 10..

[35] R Development Core Team (2008). R: A language and environment for statistical
computing..

[36] Truelsen T, Piechowski-Jóźwiak B, Bonita R, Mathers C, Bogousslavsky J (2006). Stroke incidence and prevalence in Europe: a review of available
data.. Eur J Neurol.

[37] McKevitt C, Coshall C, Tilling K, Wolfe C (2005). Are there inequalities in the provision of stroke care? Analysis
of an inner-city stroke register.. Stroke.

[38] Dhamoon M, Moon Y, Paik M, Boden-Albala B, Rundek T (2009). Long-term functional recovery after first ischemic
stroke.. Stroke.

[39] Patel M, Coshall C, Rudd AG, Wolfe CD (2003). Natural history of cognitive impairment after stroke and factors
associated with its recovery.. Clin Rehab.

[40] Hackett ML, Yapa C, Parag V, Anderson CS (2005). Frequency of depression after stroke: a systematic review of
observational studies.. Stroke.

[41] Chausson N, Olindo S, Cabre P, Saint-Vil M, Smadja D (2010). Five year outcome of a stroke cohort in Martinique, French West
Indies.. Stroke.

