# Supplementary figures and images for: Estimates of Outcomes Up to Ten Years after Stroke: Analysis from the Prospective South London Stroke Register
Source: PLoS Med. 2011 May 17;8(5):e1001033. doi: 10.1371/journal.pmed.1001033 (PMC3096613; doi:10.1371/journal.pmed.1001033)

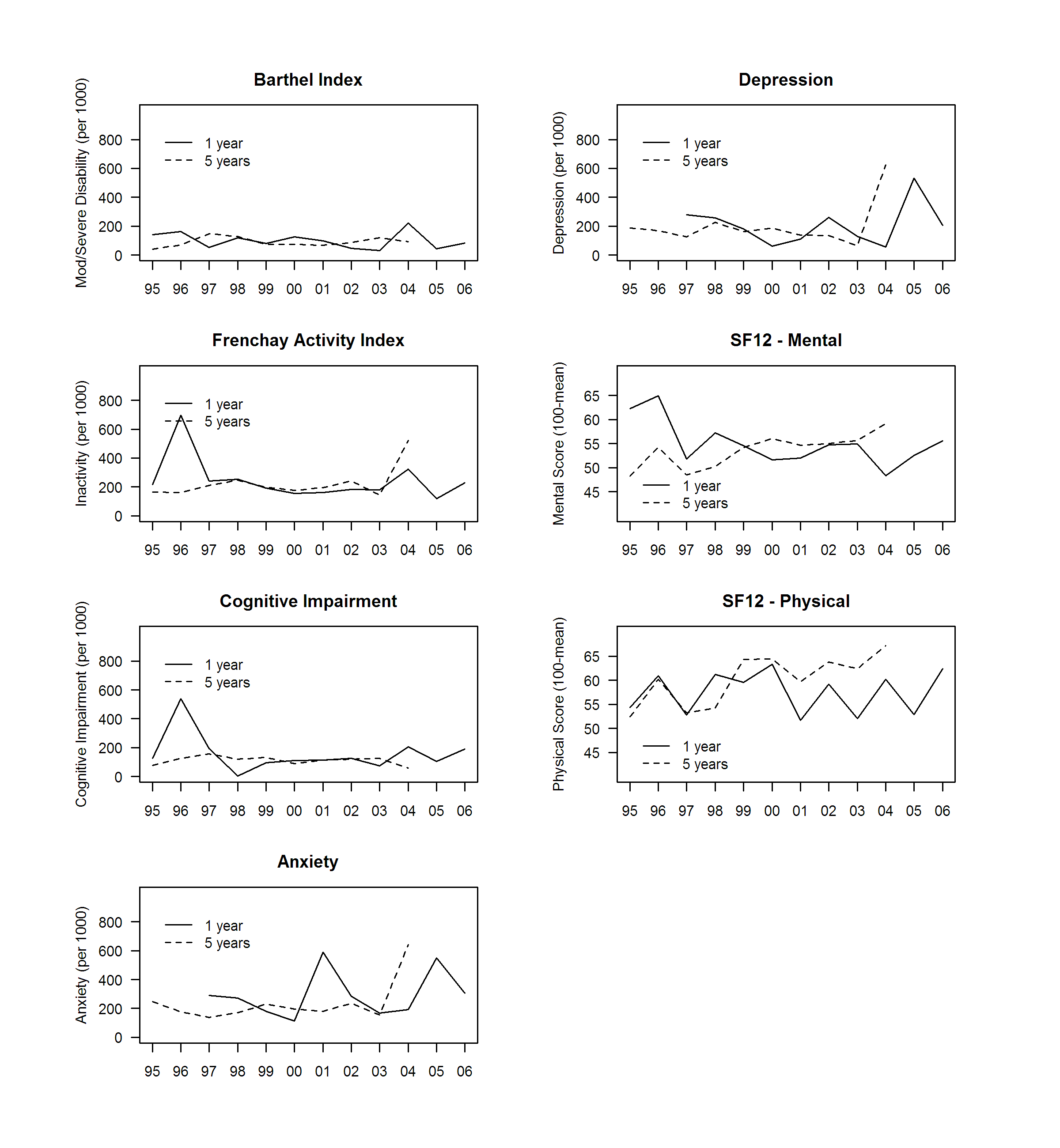

Supplement: Figure S1 — Observed rates of outcomes at 1 and 5 y after stroke by year of stroke. (TIFF) [file pmed.1001033.s001.tif]

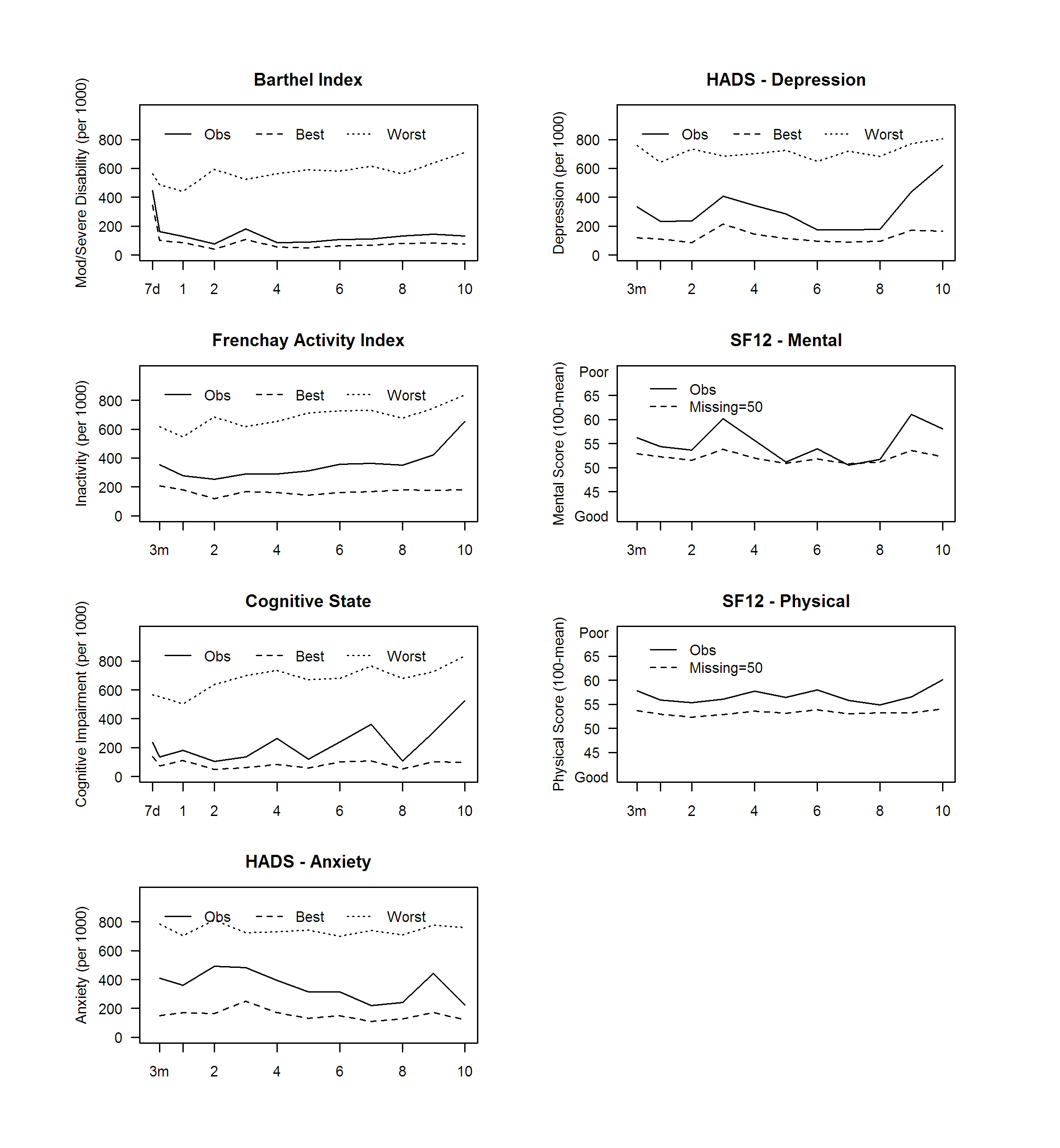

Supplement: Figure S2 — Observed age-adjusted rates of outcomes and estimated rates using imputation in survivors who were lost to follow-up. (TIFF) [file pmed.1001033.s002.tif]

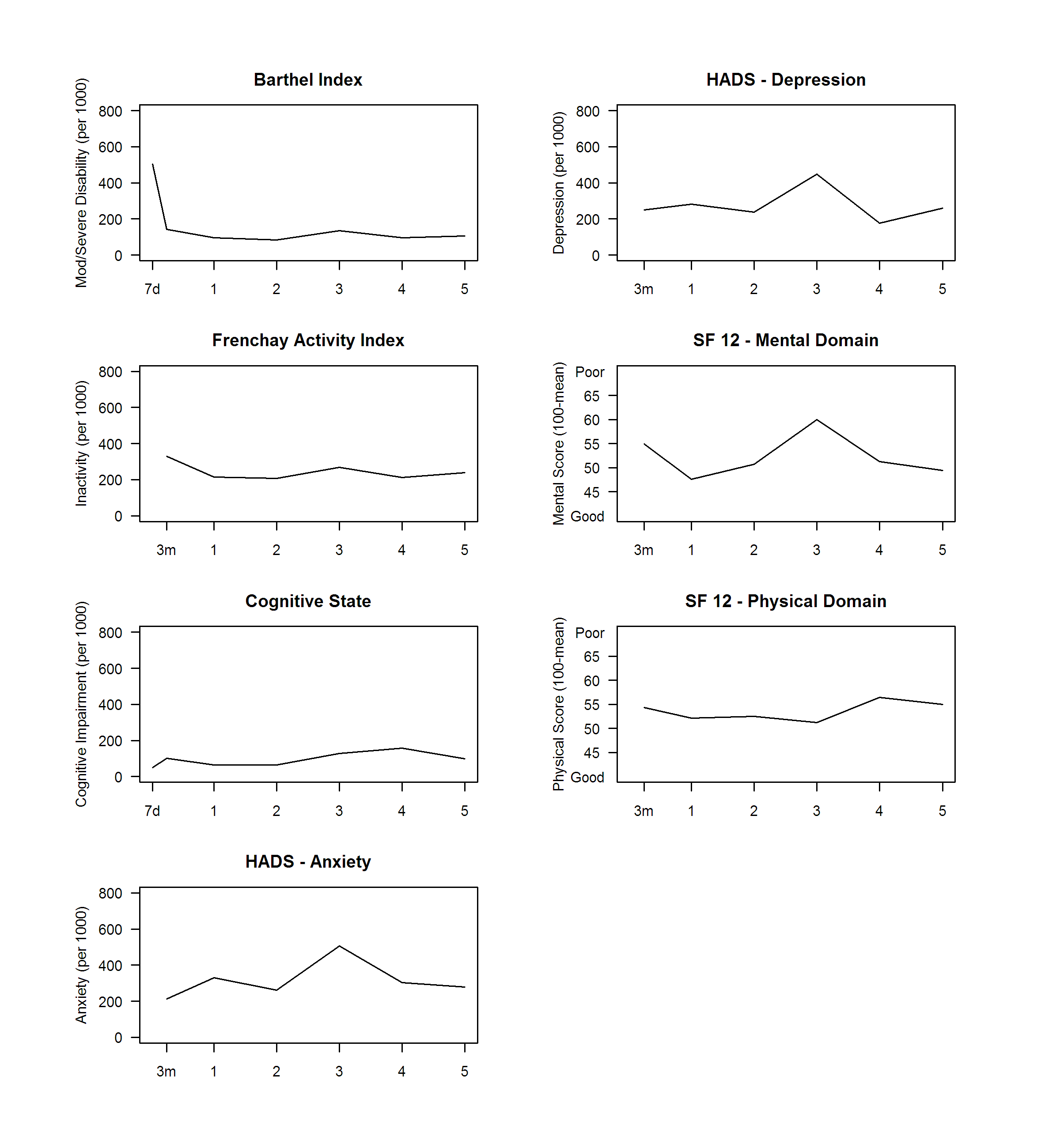

Supplement: Figure S3 — Age-adjusted rates of outcomes per 1,000 survivors with complete data up to 5 y after stroke. (TIFF) [file pmed.1001033.s003.tif]
